# Glutamate-pantothenate pathway promotes antibiotic resistance of *Edwardsiella tarda*

**DOI:** 10.3389/fmicb.2023.1264602

**Published:** 2023-09-13

**Authors:** Bei-bei Yan, Xue-sa Dong, Jun-peng Wang, Xiao-ying Li, Li An, Xi-rong Wang, Long-gang Zhang, Qing-lei Meng, Chao Wang

**Affiliations:** ^1^Department of Neonatology, Children’s Hospital Affiliated to Shandong University, Jinan, China; ^2^Department of Neonatology, Jinan Children’s Hospital, Jinan, China; ^3^Department of Genetics and Breeding, Shandong Freshwater Fisheries Research Institute, Jinan, China

**Keywords:** antibiotic resistance, *Edwardsiella tarda*, glutamate, glutathione, chloramphenicol, pantothenate

## Abstract

Although cellular metabolic states have been shown to modulate bacterial susceptibility to antibiotics, the interaction between glutamate (Glu) and chloramphenicol (CAP) resistance remains unclear because of the specificity of antibiotics and bacteria. We found that the level of Glu was upregulated in the CAP-resistant strain of *Edwardsiella tarda* according to a comparative metabolomics approach based on LC–MS/MS. Furthermore, we verified that exogenous metabolites related to Glu, the tricarboxylic acid (TCA) cycle, and glutathione (GSH) metabolism could promote CAP resistance in survival assays. If GSH metabolism or the TCA cycle is inhibited by L-buthionine sulfoximine or propanedioic acid, the promotion of CAP resistance by Glu in the corresponding pathway disappears. According to metabolomic analysis, exogenous Glu could change pantothenate metabolism, affecting GSH biosynthesis and the TCA cycle. These results showed that the glutamate-pantothenate pathway could promote CAP resistance by being involved in the synthesis of GSH, entering the TCA cycle by direct deamination, or indirectly affecting the metabolism of the two pathways by pantothenate. These results extend our knowledge of the effect of Glu on antibiotic resistance and suggest that the potential effect, which may aggravate antibiotic resistance, should be considered before Glu and GSH administration in the clinic.

## 1. Introduction

Antibiotic resistance is currently one of the most serious threats to public health (Khare, 2021). The concern about rising levels of antibiotic resistance is accompanied by the fact that very few new antibiotics are in development, and resistance to new antibiotics appears much quicker than expected. Various resistance mechanisms have been identified, including mutations in drug targets, increased expression of efflux pumps, and the synthesis of enzymes that inactivate antibiotics (Walsh, 2000). Although most antibiotic resistance studies have focused on genomic mutations and transcriptional regulation, the increased availability of high-throughput technologies has enabled the analysis of intracellular metabolites of bacterial responses to antibiotic stress (Su et al., 2015; Zhang et al., 2019; Li et al., 2020). Several studies have revealed that alterations in cellular metabolic states play an important role in modulating bacterial susceptibility to antibiotics (Peng et al., 2015a; Du et al., 2017; Zhao et al., 2021).

Metabolic perturbations alter antibiotic lethality through several processes, such as central carbon metabolism, cellular respiration, and purine metabolism (Allison et al., 2011; Peng et al., 2015b; Zhao et al., 2021). The tricarboxylic acid (TCA) cycle and oxidative phosphorylation are central metabolic processes directly disturbing the redox state *via* reactive oxygen species (ROS) byproducts (Kohanski et al., 2010). However, ROS have shown completely different characteristics for different bacteria and antibiotics, including enhanced antibiotic lethality, promoted resistance, or irrelevant effects (Ricci et al., 2012; Rosato et al., 2014; Su et al., 2018; Ye et al., 2018; Zhang et al., 2020). To eliminate the negative effects of ROS, organisms use glutathione (GSH), an important antioxidant, to maintain reduction potential in the cytoplasm. In eukaryotic cells and gram-negative bacteria, GSH is composed of three amino acids: L-glutamate (Glu), L-cysteine, and glycine (Smirnova and Oktyabrsky, 2005), and has been reported to affect bacterial sensitivity to antibiotics. Similar to the multiple characteristics of ROS, GSH exhibits antibacterial activity synergized with aztreonam, carbenicillin, ceftazidime, and meropenem against *Acinetobacter baumannii*, *Pseudomonas aeruginosa*, and *E. coli* (Goswami and Jawali, 2007; Schairer et al., 2013; Alharbe et al., 2017) and antagonizes the ability to promote fluoroquinolone resistance in *E. coli* (Goswami et al., 2006). Furthermore, metabolites related to the TCA cycle, such as α-ketoglutarate, succinate, malate, and NADH, can enhance CAP resistance (Wang et al., 2021). These studies demonstrate that the influences of the TCA cycle and redox state are complex and that these compounds disturb bacterial survival through various processes that are not yet fully understood.

In this study, we aimed to explore how the glutamate-related pathway affected the bactericidal activity of CAP based on metabolomic analysis and a survival assay with the exogenous metabolites, as well as to investigate the relationship between Glu and the TCA cycle, GSH metabolism, and pantothenate under CAP stress. We found that glutamate and GSH pathway-related metabolites could promote resistance to several antibiotics; therefore, glutamate and GSH administration should be conducted more cautiously in conditions of bacterial infection.

## 2. Materials and methods

### 2.1. Bacterial strains and culture conditions

*Edwardsiella tarda* ATCC 15947 was obtained from the China Center of Industrial Culture Collection. The CAP-resistant strain (Et-Re) was derived from a CAP-susceptible strain of *E. tarda* ATCC 15947, as described previously (Wang et al., 2021). Briefly, the original strain of *E. tarda* ATCC 15947 (Et-Ori) was cultivated in Tryptic Soy Broth (TSB) medium (Solarbio Science & Technology Co., Ltd., Beijing, China) with increased concentrations of CAP (Solarbio Science & Technology Co., Ltd., Beijing, China) at 37°C for 50 passages. After subculturing, the bacteria were transferred onto a TSB agar plate at 37°C overnight, and colonies were randomly selected to measure the minimum inhibitory concentration (MIC). We selected the colony with the highest MIC value as the antibiotic-resistant strain. Both the original and CAP-resistant strains were identified by 16S RNA sequencing.

### 2.2. Metabolomic analysis

The procedures for sample preparation, metabolomics data acquisition, and analysis were derived from previous metabolomics information (Wang et al., 2021). Briefly, equivalent cells were quenched with 60% (v/v) cold methanol (Sigma) at −40°C for 5 s and collected by centrifugation at 1000 ×*g* at 4°C for 10 min. After being resuspended by 0.1 mL of pre-cooled methanol at −20°C and incubation for 60 min, the samples were collected by centrifugation at 14,000 ×*g* for 15 min, and the supernatants were transferred into a fresh microcentrifuge tube and dried in a centrifugal evaporator. The dried metabolite pellets were re-dissolved in 80% methanol and analyzed using LC–MS/MS.

LC–MS/MS analyses were performed using a Vanquish UHPLC system (Thermo Fisher) coupled with an Orbitrap Q Exactive HF-X mass spectrometer (Thermo Fisher), and the parameters were set up as previous study before (Wang et al., 2021). The raw data of MS files were processed with the Compound Discoverer software (version 3.0, Thermo Fisher Scientific). After removed the background ions and normalized by QC samples, the identification and quantitative results of peaks were used for subsequent statistical analysis. In this study, metabolomics data related to Glu pathway was reanalysis. The metabolites with *p*-value <0.05 and log2|(FoldChange)| > 1 were defined as differential metabolites. Glu pathway was annotated by the KEGG database^1^ and Pathview (Luo and Brouwer, 2013; Luo et al., 2017), and the scatter diagram was drawn by the relative content of metabolites normalized with the original strain.

### 2.3. Antibiotic bactericidal assays

Bacterial cells were cultured in TSB medium overnight at 37°C and collected by centrifugation at 5000 ×*g* for 5 min. The collected cells were washed three times with sterile saline and diluted to 10^6^ CFU/mL with M9 minimal media containing 1 mM MgSO_4_ and 0.1 mM CaCl_2_. The metabolites and/or CAP were added to the medium and incubated at 37°C for 5 h. The concentrations of CAP for Et-Ori and Et-Re were 100 mg/L and 2000 mg/L, respectively, while the concentration of the metabolites was 10 mM. Finally, 10 μL of the culture was removed, diluted, plated on TSB agar plates, and incubated at 37°C for 18 h. The colonies were counted, and the CFU/mL was calculated.

### 2.4. Metabolome analysis of exogenous addition of Glu

Bacterial cells cultured overnight in TSB medium were harvested, washed with sterile saline, and diluted with M9 minimal medium as described above. Exogenous Glu was added to M9 medium at a concentration of 10 mM and incubated at 37°C for 5 h. The bacterial cells were harvested rapidly by centrifugation at 5000 ×*g* for 5 min. After washing twice with cold, sterile saline, the bacterial cells were frozen in liquid nitrogen and stored at −80°C. Metabolomic analysis was performed as described previously.

### 2.5. Survival assay of four antibiotics

Bacterial culture conditions were the same as those used for the survival assay. Briefly, the culture was incubated overnight and diluted to 5 × 10^5^ CFUs/mL. The concentrations of kanamycin (KAN), ceftazidime (CAZ), ciprofloxacin (CIP), and meropenem (MEM) were 10 mg/L, 0.2 mg/L, 3 μg/L, and 0.2 mg/L, respectively. The concentrations of Glu and GSH were 10 mM. Bacteria with Glu/GSH and antibiotics were cultured in a 96-well plate, and the control was cultured with the corresponding antibiotic only. After incubation for 5 h, cell densities were determined using an optical density of 600 nm (OD600). The viability fold was calculated by dividing the OD600 of the experimental group by the control.

## 3. Results

### 3.1. Analysis of different metabolites and KEGG pathway of CAP resistance strain

**Figure 1:**
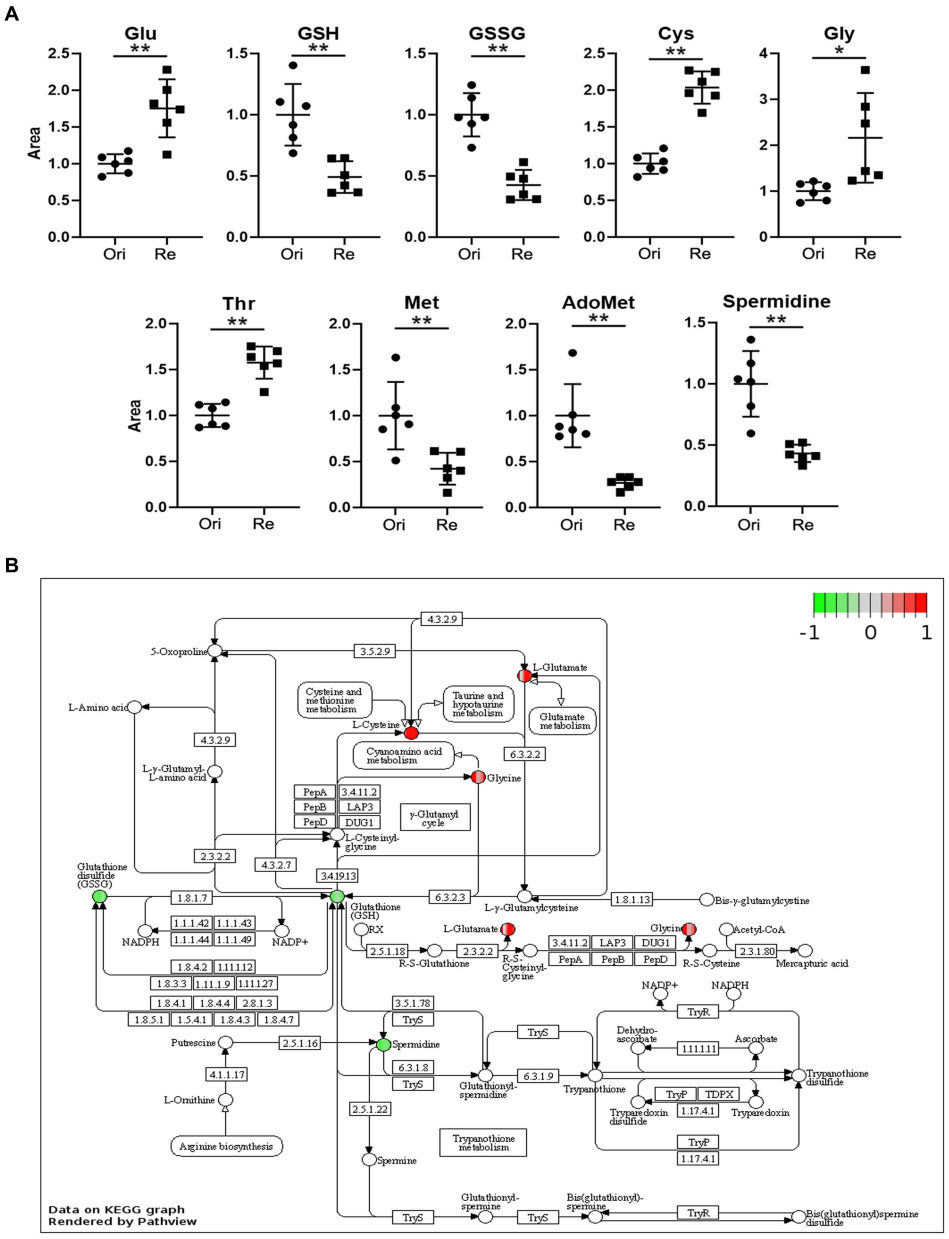
The scatter diagrams of the metabolites related to Glu metabolism **(A)** and GSH pathway of different metabolites in the CAP-resistant strain **(B)**. The groups Ori and Re represent the original and CAP-resistant strains, respectively. The y-axis represents the relative area of the metabolite compared with the original strain, which was normalized to 1.0 (*/**, *p* < 0.05/0.01, compared with the original group). The different metabolites with increased or decreased are shown in red or green, and the graph is rendered by KEGG Pathview (https://pathview.uncc.edu). Glutamate (Glu), Glutathione (GSH), Glutathione disulfide (GSSG), Cysteine (Cys), Glycine (Gly), Threonine (Thr), Methionine (Met), and Adenosylmethionine (AdoMet).

In previous studies, we compared metabolites of Et-Re with Et-Ori *via* high-performance LC–MS/MS, and 364 different metabolites and 12 different pathways were identified (Wang et al., 2021). In this study, we focused on different metabolites related to the Glu and GSH pathway, including Glu, GSH, glutathione disulfide (GSSG), cysteine (Cys), glycine (Gly), threonine (Thr), methionine (Met), adenosylmethionine (AdoMet), and spermidine (Figure 1A). The relative contents of Glu, Cys, Gly, and Thr increased in Et-Re, while those of the remaining five metabolites decreased. In the KEGG enrichment pathway of GSH metabolism, the increased metabolites of Glu, Gly, and Cys are shown in the red cycle, and the decreased metabolites of GSH, GSSG, and spermidine are shown in the green cycle (Figure 1B).

### 3.2. Metabolites related to the Glu and GSH pathways promote CAP resistance

**Figure 2:**
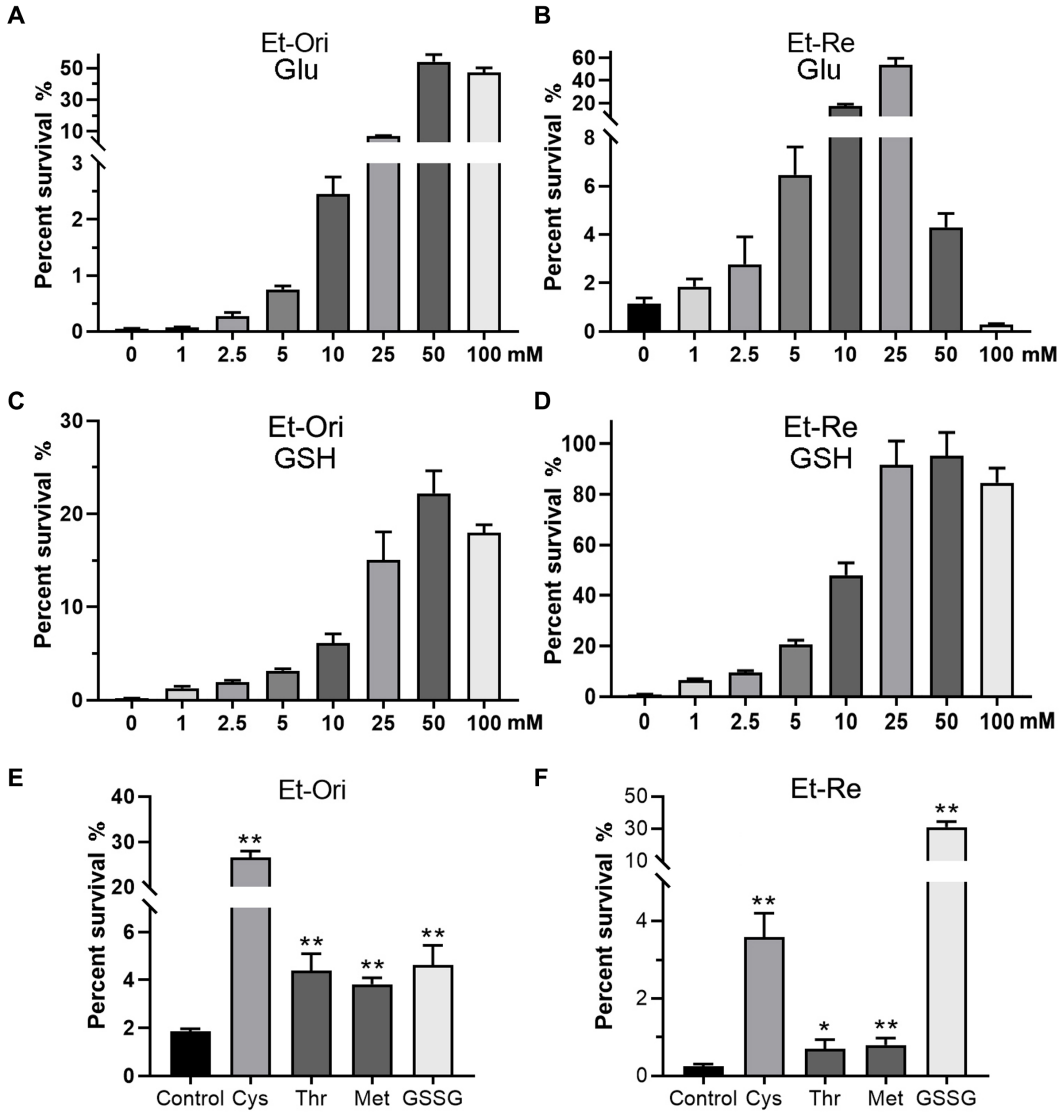
Metabolites related to the Glu and GSH pathways promote CAP resistance. Glu **(A,B)**, GSH **(C,D)**, and other metabolites **(E,F)** increased the viability of Et-Ori and Et-Re cells against CAP. The x-axis represents the different concentrations of Glu **(A,B)** or GSH **(C,D)**. The concentrations of Cys, Thr, Met, and GSSG were 10 mM. The control group contained CAP without metabolites **(E,F)**, whereas the other groups contained CAP and metabolites (*/**, *p* < 0.05/0.01, compared with the control). The y-axis represents the percent survival of *E. tarda*.

The percent survival of Et-Ori and Et-Re depended on the concentrations of Glu and GSH under CAP pressure in the M9 medium. As the concentration of Glu increased gradually, the viabilities of the two groups increased, with a peak concentration of 50 mM in Et-Ori and 25 mM in Et-Re (Figures 2A,B). However, the percent survival declined rapidly when the concentration of Et-Re was more than 50 mM. GSH gradually increased the viability of Et-Ori and Et-Re as the concentration increased, and both had a peak concentration of 50 mM (Figures 2C,D).

CAP resistance could be promoted by the other four metabolites related to the Glu and GSH pathway, including Cys, Thr, Met, and GSSG (Figures 2E,F). The highest percent survival in Et-Ori was in the Cys group, a 14.2-fold increase compared with the control. In Et-Re, Cys and GSSG promoted CAP resistance with 14.8- and 128.2-fold survival percentages, respectively.

### 3.3. GSH metabolism is important in CAP resistance

**Figure 3:**
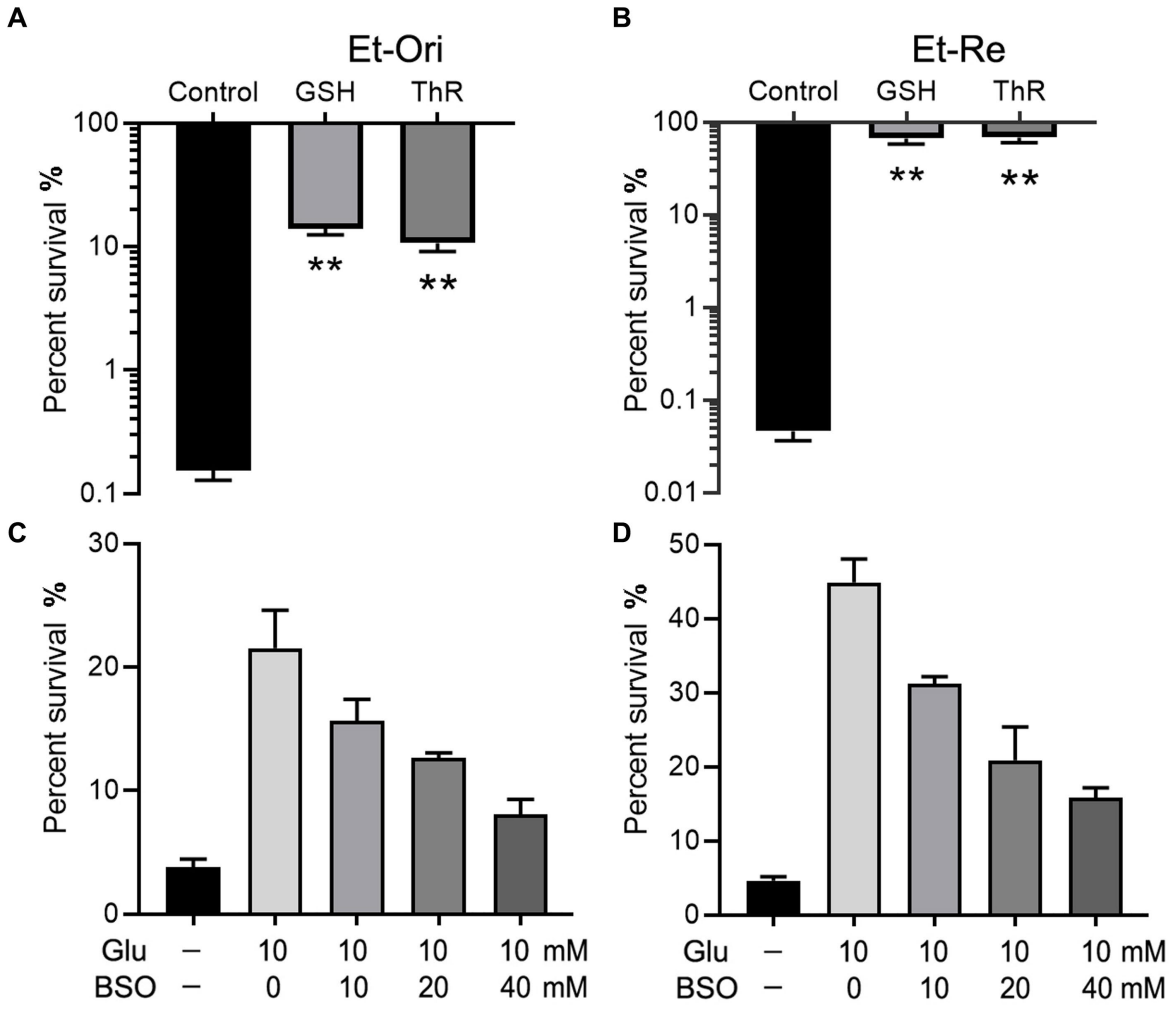
Glu and GSH metabolism affect CAP resistance of Et-Ori **(A,C)** and Et-Re **(B,D)**. The concentrations of GSH and thiourea (ThR) were 10 mM. The control group contained CAP without metabolites, whereas the others contained CAP and metabolites **(A,B)** (**, *p* < 0.01 compared with the control). The concentrations of Glu and BSO are shown on the x-axis (**C,D**, respectively).

GSH is an important intracellular reductant; therefore, we used thiourea (ThR) to verify whether reductants promote CAP resistance. Similar to GSH, which could increase the survival rate by 90.0 folds, ThR dramatically promoted CAP resistance in the M9 medium with a 69.0-fold increase in Et-Ori (Figure 3A). Furthermore, the viabilities of Et-Re were substantially improved by GSH and ThR, with 1,458- and 1,483-fold increases, respectively (Figure 3B). L-Buthionine sulfoximine (BSO) is an inhibitor of glutamylcysteine synthetase, which is involved in glutathione synthesis. With increasing concentrations of BSO, the viabilities gradually decreased in Et-Ori and Et-Re, and Glu could no longer promote CAP resistance (Figures 3C,D).

### 3.4. Glu affects CAP resistance through the TCA cycle

**Figure 4:**
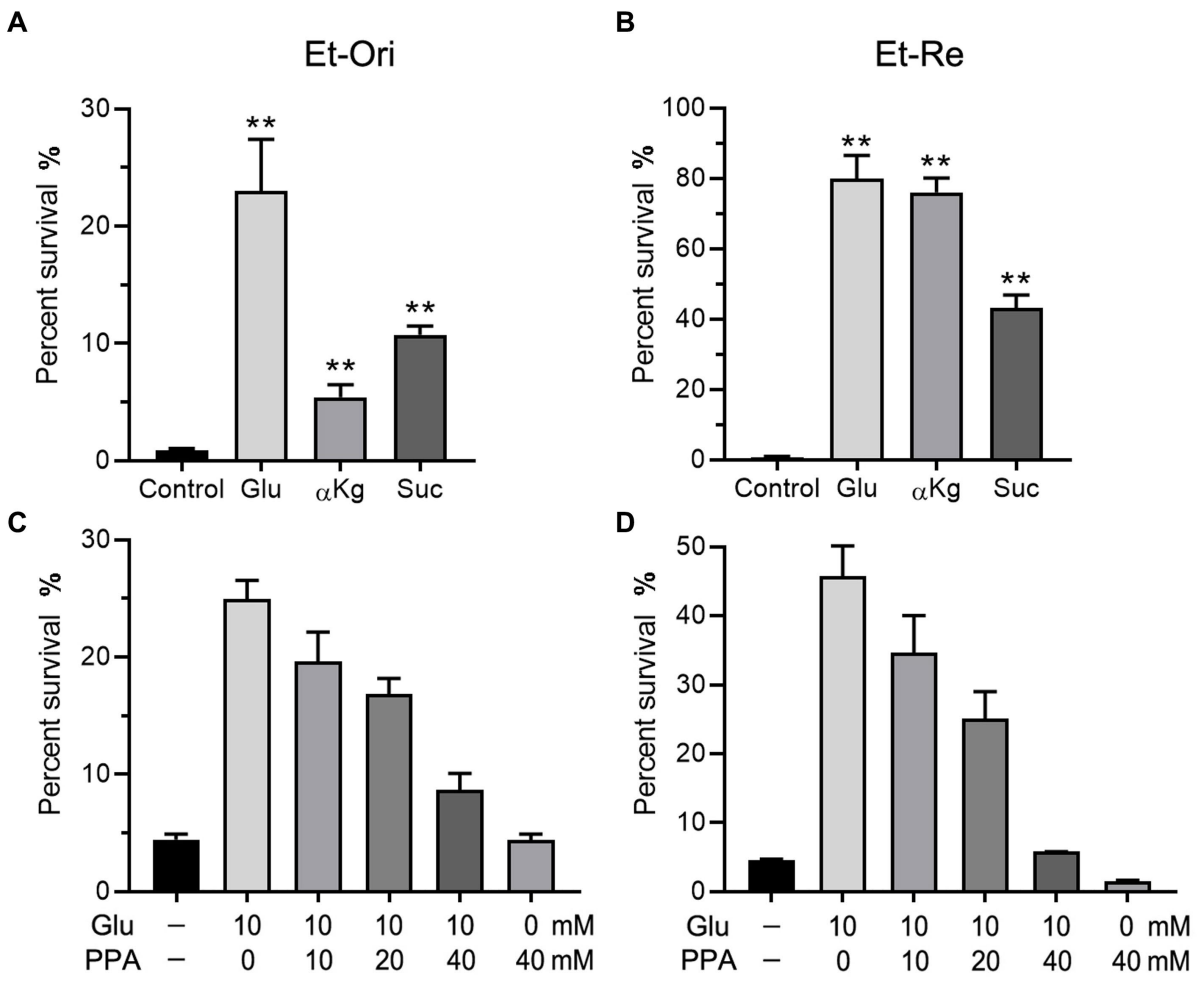
Glu and the TCA cycle affect CAP resistance of Et-Ori **(A,C)** and Et-Re **(B,D)**. The concentrations of Glu, α-ketoglutarate (αKg), and succinate (Suc) were 10 mM. The control group contained CAP without metabolites, whereas the others contained CAP and metabolites **(A,B)** (**, *p* < 0.01 compared with the control). Concentrations of Glu and propanedioic acid (PPA) are shown on the x-axis **(C,D)**.

Glu can be converted to α-ketoglutarate (αKg) and enter the TCA cycle. According to a previous study (Wang et al., 2021), metabolites in the TCA cycle can increase CAP resistance in *E. tarda*. Glu and the metabolites in the TCA cycle promoted the differences in viability. The percent survival of the Glu group was the highest in the Et-Ori and Et-Re groups (Figures 4A,B). The αKg group had a lower survival rate than the Suc group in Et-Ori, but the opposite was true for Et-Re (Figures 4A,B). Propanedioic acid (PPA) is a competitive inhibitor of succinate dehydrogenase, which could disturb the CAP resistance metabolites provided in the TCA cycle (Wang et al., 2021). Similar to BSO, the survival rates gradually decreased in Et-Ori and Et-Re with increasing concentrations of PPA (Figures 4C,D).

### 3.5. Analysis of different metabolites and KEGG pathway generated by exogenous glutamate

**Figure 5:**
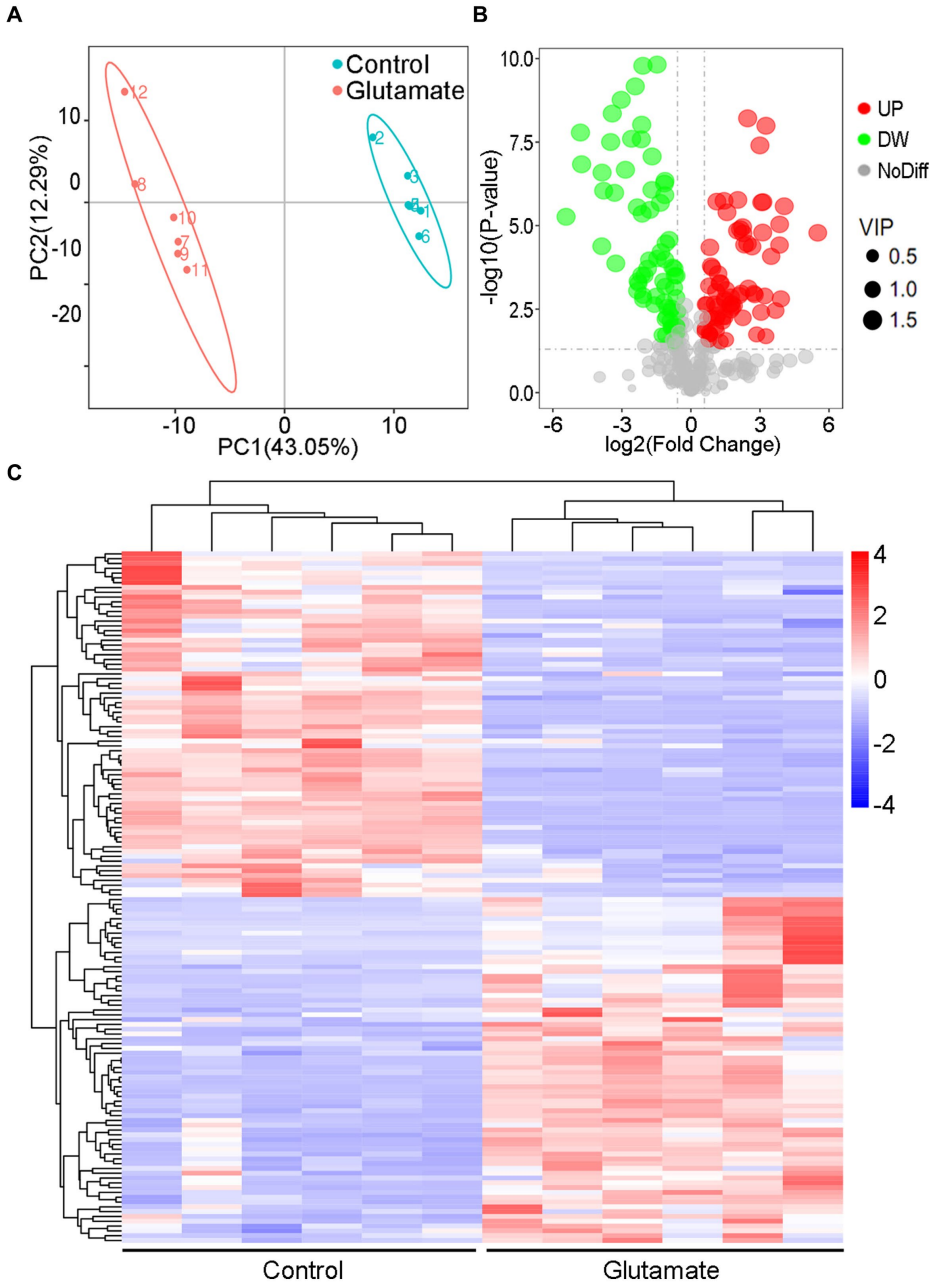
Metabolomic analysis of the different metabolites. **(A)** PLSDA in the control and exogenous glutamate groups. Each dot represents a biological replicate. **(B)** Volcano plot of different metabolites identified between the control and exogenous glutamate groups. The X and Y axes represent the fold change in metabolites and statistical significance in the two samples, respectively. The green, red, and gray dots represent the level of the metabolites significantly downregulated, and upregulated, and with no significant difference, respectively. **(C)** Heat maps of significantly different metabolites in the presence or absence of glutamate. Blue and red indicate a low and high abundance of metabolites, respectively.

**Figure 6:**
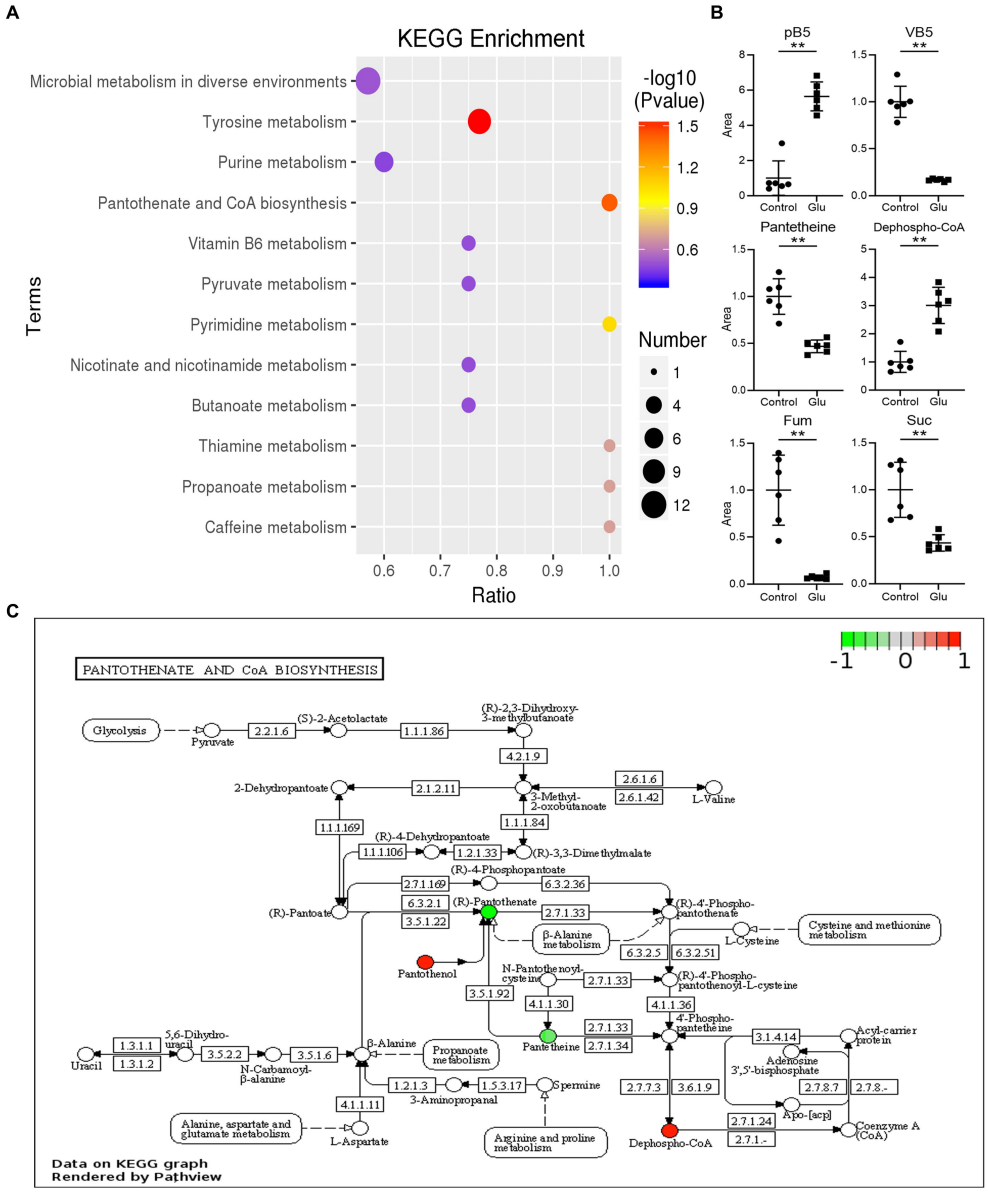
KEGG analysis of different metabolites. **(A)** List of KEGG enrichment pathways. The value of *p* (−log 10) of the enrichment pathway is represented by color (red to blue), and the number of significantly different metabolites is shown by diameter. The x-axis represents the ratio of significantly different metabolites divided by the total metabolites in the pathway. **(B)** Scatter diagrams of metabolites with significant differences in related pathway. The y-axis is the relative area of the metabolite compared to the control group, which is normalized to 1.0 (**, *p* < 0.01 compared with the control group). **(C)** The significantly enriched pathway related to VB5 in the exogenous glutamate group compared with the control group. Green and red represent decreased and increased metabolite levels, respectively.

The metabolites in the exogenous glutamate and control groups were measured by LC–MS/MS. The two groups were completely separated using the PLSDA (Figure 5A). There were 325 metabolites identified, and significantly different metabolites were 144 in the Glu group compared with the control group. There were 72 metabolites with both upregulated and downregulated (Figure 5B). The abundance of metabolites significantly differed between the two groups (Figure 5C). According to KEGG enrichment analysis, there were two significantly different pathways (*p* < 0.05; Figure 6A): tyrosine metabolism and pantothenate and CoA biosynthesis. The pantothenate pathway has four significantly different metabolites: provitamin B5 (pB5), dephospho-CoA, pantothenate (VB5), and pantetheine. The first two metabolites had upregulated, and the latter two had downregulated (Figures 6B,C). The level of the two metabolites of the TCA cycle, fumarate and succinate, were both downregulated (Figure 6B), but the TCA cycle was not significantly changed.

### 3.6. Metabolites related to VB5 altered bactericidal ability of CAP

**Figure 7:**
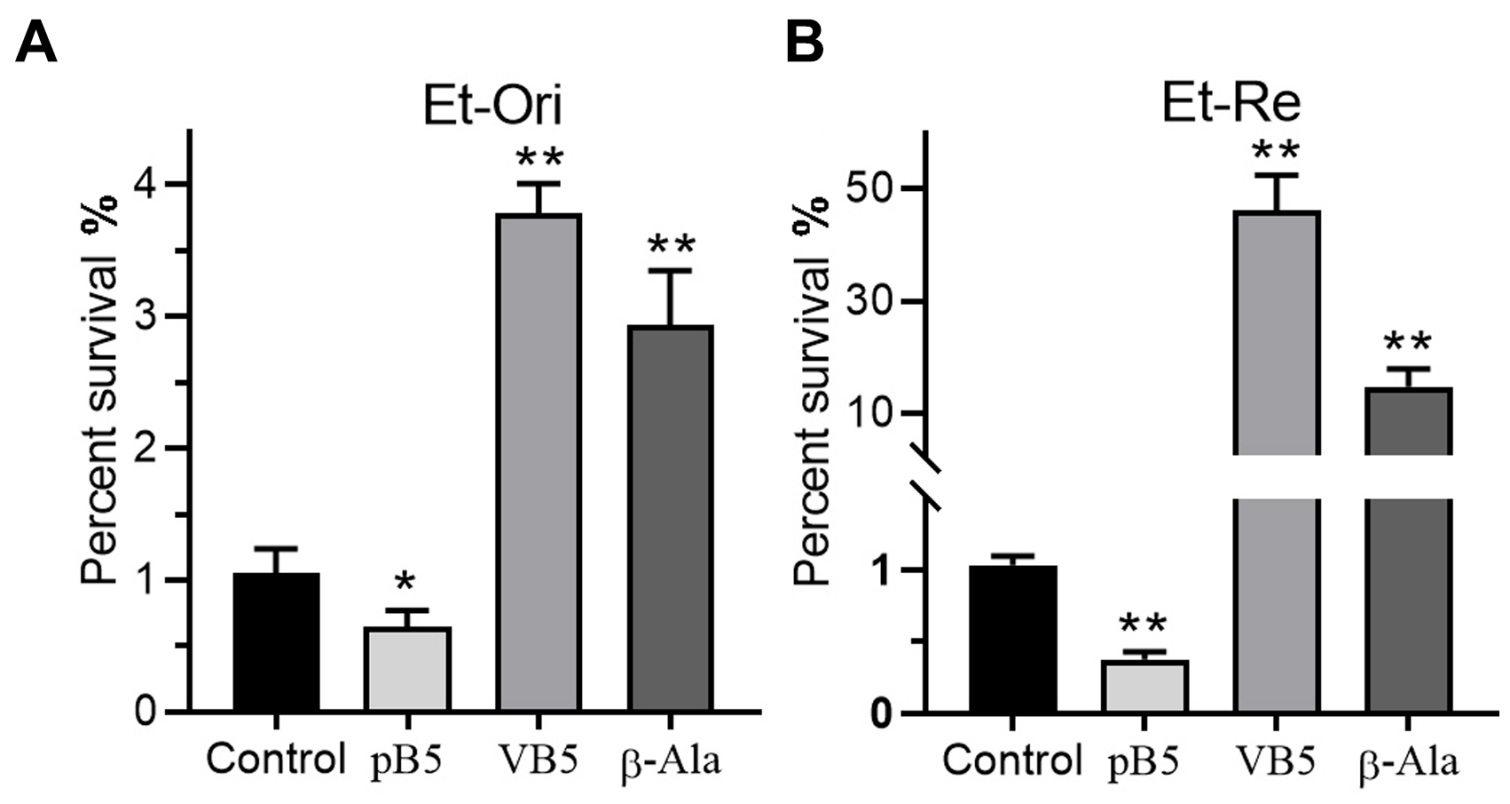
Metabolites related to VB5 affect CAP resistance of Et-Ori **(A)** and Et-Re **(B)**. The concentrations of provitamin B5 (pB5), pantothenate (VB5), and β-alanine (β-Ala) were 10 mM. The control group contained CAP without metabolites, whereas the others contained CAP and metabolites (*/**, *p* < 0.05/0.01, compared with the control).

Metabolites in the VB5 pathway have different effects on the bactericidal efficiency of CAP. Provitamin B5 could reduce the viability of *E. tarda* under the pressure of CAP, but VB5 and β-Ala promote CAP resistance (Figures 7A,B). The percent survival was 0.61-, 3.60-, and 2.80-fold in Et-Ori and 0.36-, 44.9-, and 14.3-fold in Et-Re compared with the control. The effects of these metabolites were more pronounced in Et-Re.

### 3.7. Glu and GSH promote other antibiotic resistance

**Figure 8:**
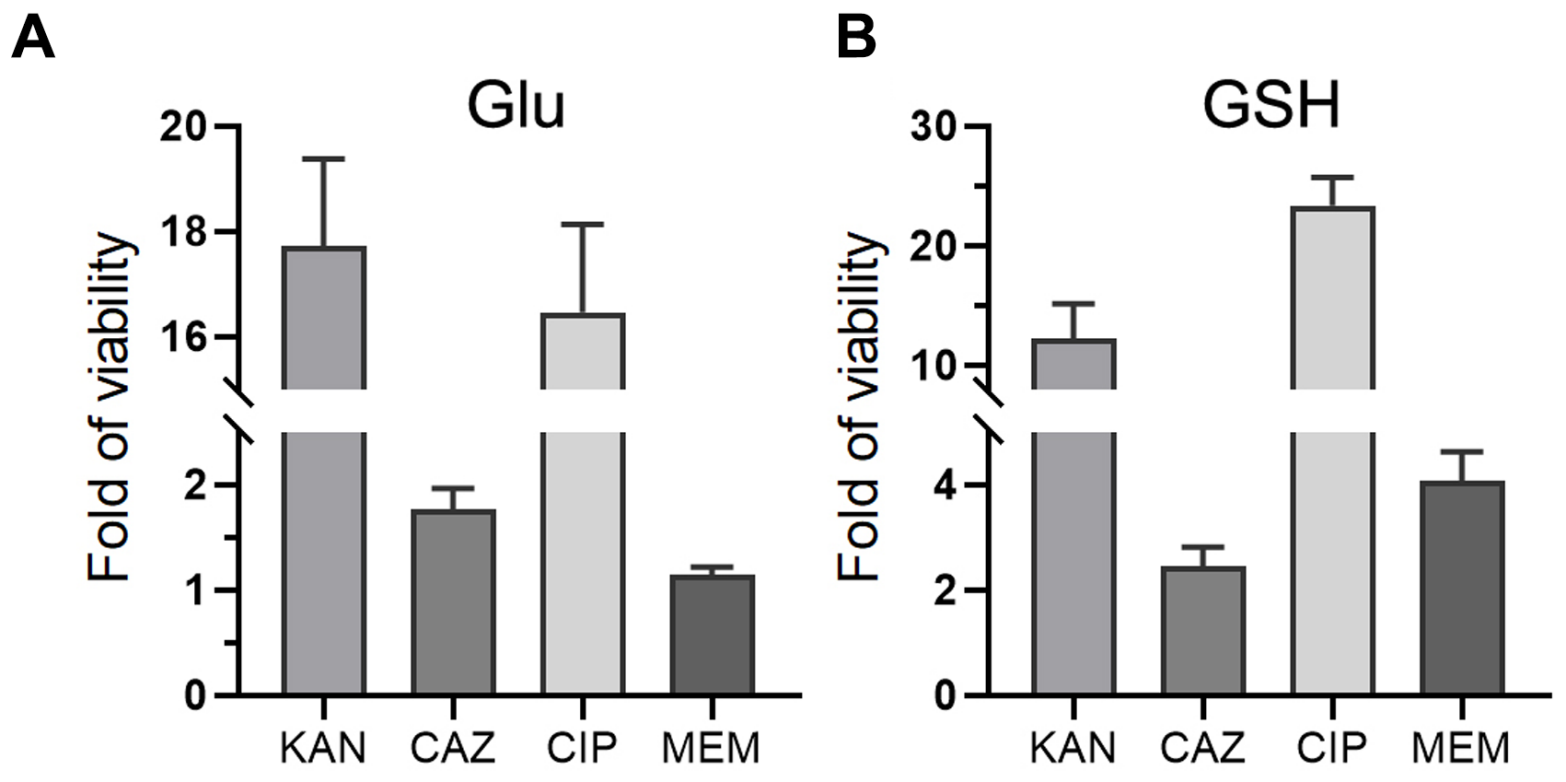
Glu and GSH affect resistance of other antibiotics. The TSB medium of experimental group contains both antibiotic and metabolite of Glu **(A)** or GSH **(B)**, while that of the control group only contains corresponding antibiotic. The y-axis is the OD_600_ of the experimental group divided by that of the control group.

To investigate whether Glu and GSH could promote bacterial resistance to other antibiotics, we added Glu or GSH with four antibiotics separately into the TSB medium, including kanamycin (KAN), ceftazidime (CAZ), ciprofloxacin (CIP), and meropenem (MEM). Glu and GSH could increase resistance to the last three antibiotics but only GSH affected MEM (Figures 8A,B). The increase in OD_600_ of the four antibiotics by Glu was 17.7-, 1.76-, 16.5-, and 1.15-fold (Figure 8A), and the increase in GSH was 12.2-, 2.45-, 23.4-, and 4.09-fold (Figure 8B).

## 4. Discussion

Mounting evidence has revealed that the cellular metabolic state is important in modulating antibiotic susceptibility in multiple bacterial species (Van Acker et al., 2014). In our previous study, metabolomic analysis of *E. tarda* showed that Glu-related metabolism was significantly different in CAP resistance strains (Wang et al., 2021). Subsequently, we found that the percent survival of *E. tarda* increased as the concentration of Glu increased, demonstrating that Glu could affect the bactericidal ability of CAP. Glutamate is not only an amino acid but is also involved in many important metabolic processes. Glutamate metabolism is closely related to GSH metabolism and the TCA cycle. The synthesis of GSH starts with the combination of Glu and Cys to γ-L-glutamyl-L-cysteine catalyzed by γ-glutamylcysteine synthetase and consumes ATP. In addition, glutamate can be converted to α-ketoglutarate by deamination; therefore, glutamate is a key intermediary between α-amino acids and the TCA cycle.

GSH is an important reductant that protects cells against oxidative stress. Numerous studies have demonstrated that the intracellular redox state is lethal to bacteria under antibiotic stress (Kohanski et al., 2008; Davies et al., 2009; Foti et al., 2012; Vilcheze et al., 2013). However, the effects of GSH on the bactericidal efficiency of antibiotics have been inconsistent. On the one hand, GSH increases the sensitivity of *E. coli* and *P. aeruginosa* to trimethoprim and ciprofloxacin by inhibiting the expression of *acrAB-tolC* and disrupting biofilms, respectively (Klare et al., 2016; Song et al., 2021). On the other hand, GSH inactivates fosfomycin by opening the epoxide ring of the antibiotics (Allocati et al., 2009). GSH can protect *E. coli* against fluoroquinolones (ciprofloxacin, norfloxacin, ofloxacin, and gatifloxacin) by disturbing ROS, but not against nonfluoroquinolones (ampicillin and tetracycline) (Goswami et al., 2006). In this study, we found that ThR provided a similar percent survival to GSH, which showed that the reductant could promote CAP resistance. The increased percent survivals of Glu and GSH were positively correlated with their respective concentrations. Metabolites related to GSH metabolism, such as Cys, Met, and GSSG, could promote CAP resistance. Cys is a component of GSH, and Met is the provider of thiol. More importantly, if GSH metabolism is disturbed by the inhibitor of glutamylcysteine synthetase, Glu can no longer promote CAP resistance. As a component of GSH, the effect of Glu on CAP resistance may be achieved by regulating GSH synthesis.

As an important central carbon and energy metabolism pathway, the TCA cycle is associated with antibiotic resistance through its effect on ROS (Su et al., 2018). It has been demonstrated that perturbation of the TCA cycle could reduce antibiotic sensitivity (Thomas et al., 2013), whereas promotion of the TCA cycle restores the susceptibility of bacteria to antibiotics (Peng et al., 2015b). Furthermore, lower basal respiration can prevent metabolic toxicity and minimize drug lethality (Lopatkin et al., 2021). In a previous study, we demonstrated that metabolites in the TCA cycle, such as α-ketoglutarate, succinate, fumarate, malate, and oxaloacetate, could increase CAP resistance of *E. tarda* (Wang et al., 2021). In this study, we found that the percent survival after adding Glu was higher than that of metabolites in the TCA cycle. Furthermore, the increased survival provided by Glu gradually disappeared if the TCA cycle was disturbed by the increased concentration of the competitive inhibitor of succinate dehydrogenase.

Inhibition assays of glutamylcysteine synthetase and succinate dehydrogenase indicated that Glu promoted CAP resistance by directly affecting the synthesis of GSH and the TCA cycle. In addition, the analysis of the exogenous Glu effect on metabolomics showed that Glu could affect CAP resistance *via* VB5. Pantothenate and CoA biosynthesis was the significantly enriched pathway. Catalyzed by aspartate transaminase, Glu can be converted to aspartate, and the latter resolves into CO2 and β-alanine catalyzed by aspartate decarboxylase to enter pantothenate metabolism. β-Alanine is indispensable for synthesizing pantothenate, as it is a precursor of coenzyme A (CoA), which participates in converting pyruvate to acetyl-CoA for the TCA cycle. Both β-alanine and pantothenate promoted CAP resistance, verifying that the pantothenate pathway played an important role in CAP resistance. On the one hand, Pantothenol (pB5) is a provitamin of pantothenate, an inhibitor of the phosphorylation activity of the prokaryotic pantothenate kinase, which catalyzes the first step of the coenzyme A biosynthetic pathway (Chohnan et al., 2014). Pantothenol decreased the percent survival rate, which showed that sensitivity to CAP increased when the pantothenate pathway was blocked. On the other hand, pantothenate is an effective modulator of the thiol-disulfide system, which increases the antioxidant capacity and stimulates glutathione biosynthesis (Moiseenok et al., 2000; Slyshenkov et al., 2004). Furthermore, the level of the two metabolites in the TCA cycle, fumarate and succinate, were downregulated due to the influence of exogenous Glu. These two metabolites are located between αKg and oxaloacetate. The former is the node where Glu enters the TCA cycle and the latter is the end of the TCA cycle, which reacts with acetyl-CoA to restart the TCA cycle. The two metabolites were influenced by Glu and pantothenate on the TCA cycle. These results verify that the glutamate-pantothenate pathway plays an important role in CAP resistance by affecting the TCA cycle and GSH metabolism.

**Figure 9:**
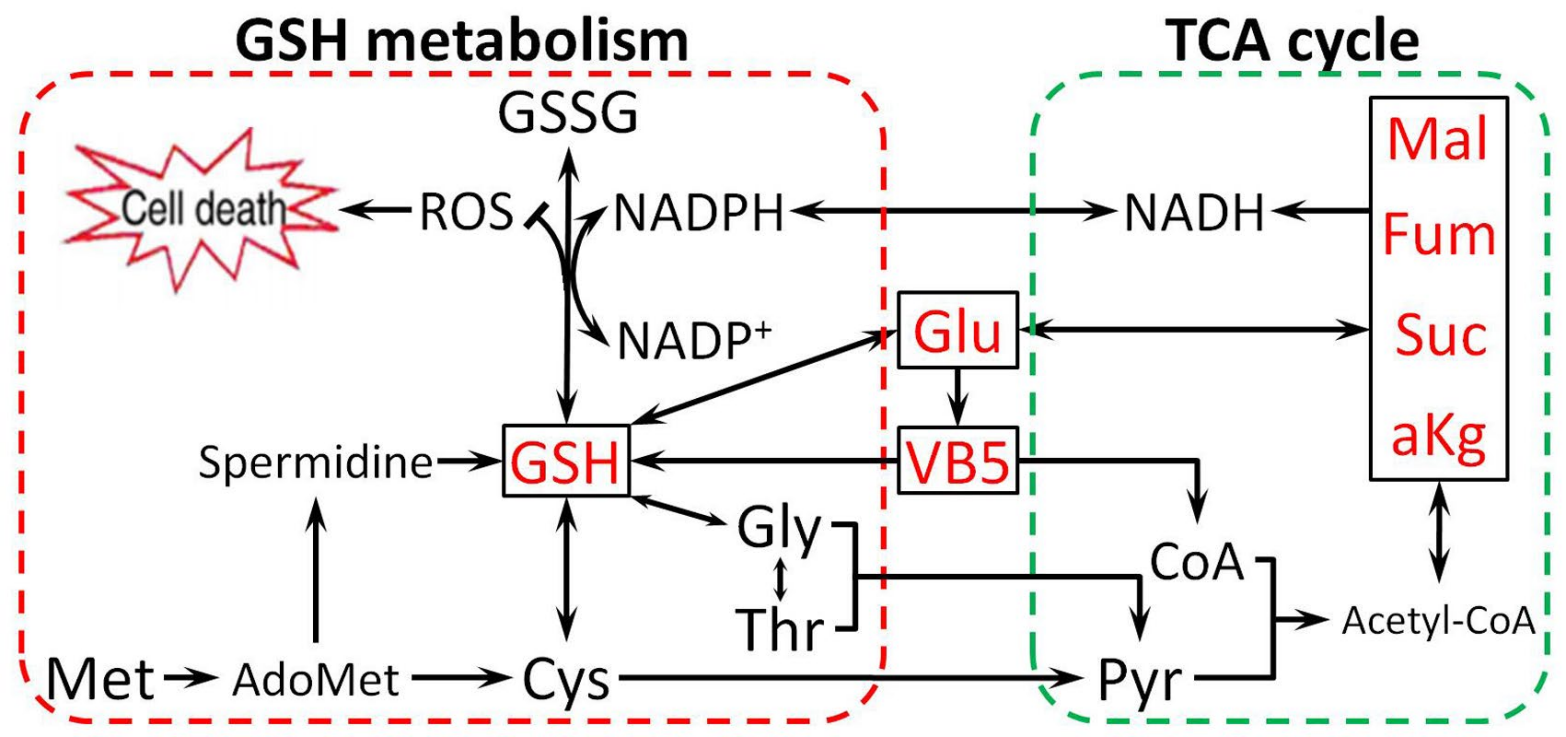
The proposed mechanism by which metabolites related to the Glu pathway impart resistance to CAP. Glu enters the tricarboxylic acid (TCA) cycle and synthesizes GSH, directly or indirectly influencing the TCA cycle and GSH metabolism by VB5. Pyruvate (Pyr), Malate (Mal), Coenzyme A (CoA), and Acetyl coenzyme A (Acetyl-CoA).

Based on the above findings, we found that Glu promotes antibiotic resistance in two ways. First, Glu is directly involved in synthesizing GSH as a raw material or enters the TCA cycle by deamination. Second, Glu can change pantothenate metabolism, which affects glutathione biosynthesis and the TCA cycle. Metabolites related to the TCA cycle could directly increase the CAP resistance of *E. tarda* (α-ketoglutarate, succinate, fumarate, malate, and oxaloacetate) or be activated as cofactors (thiamine, nicotinate, NADH, and NADPH) based on our previous study (Wang et al., 2021). NADH, primarily derived from the TCA cycle, is a hydrogen provider of NADPH (Meylan et al., 2017), which contributes to maintaining the reduction state of GSH (Deponte, 2013). The effect of Glu on CAP resistance is illustrated in Figure 9. Glutamate-pantothenate metabolism is located in the center of the pathway, and Glu is a communication bridge between GSH and the TCA cycle.

As the therapeutic effect of antibiotics is weakened by drug resistance, an effective adjuvant is required to restore the bactericidal ability of antibiotics. Fumarate has been approved for treating *Pseudomonas* infection by the U.S. Food and Drug Administration. There are dual characteristics of metabolites in the TCA cycle and GSH metabolism: restoration of susceptibility (Allison et al., 2011; Peng et al., 2015b) and increased viability (Wang et al., 2021). In particular, Glu and GSH can impair the bactericidal ability of multiple antibiotics, including CAP, KAN, CAZ, CIP, and MEM. Although GSH is suggested to inhibit the growth of *S. aureus*, *E. coli*, *Klebsiella pneumonia*, and *P. aeruginosa* (Schairer et al., 2013), metabolite adjuvants must be limited strictly to specific antibiotics and bacteria. Survival of the bacterial population provides more opportunities for rare mutations and the evolution of resistance during antibiotic therapy (Cohen et al., 2013; Levin-Reisman et al., 2017).

Herein, we found that the glutamate-pantothenate pathway promotes antibiotic resistance by affecting GSH metabolism and the TCA cycle. Our study fills some knowledge gaps in the relationship between Glu, VB5, GSH metabolism, and the TCA cycle. However, antibiotic-mediated cell death is a complex process that varies with different antibiotics and bacteria, leading to the requirement of further research to improve our understanding of the interplay between antibiotics and metabolites.

## Data availability statement

The data presented in the study are deposited in the figshare repository, Accession number 10.6084/m9.figshare.24031929.

## Author contributions

B-bY: Investigation, Writing – original draft. X-sD: Writing – review & editing, Investigation, Methodology. X-yL: Writing – review & editing, Investigation. LA: Writing – review & editing, Investigation. X-rW: Writing – review & editing, Investigation. L-gZ: Writing – review & editing, Investigation. Q-lM: Conceptualization, Writing – review & editing. CW: Conceptualization, Supervision, Writing – original draft, Writing – review & editing. J-pW: Investigation, Writing - review & editing.

## Funding

The author(s) declare financial support was received for the research, authorship, and/or publication of this article. This work was financially supported by Shandong Provincial Natural Science Foundation of China (ZR2022MC170, ZR2019PH019), Agricultural Seed Project of Shandong Province (2019LZGC013), and Innovation Project of Science and Technology of Shandong Province (2021CXGC010806).

## Conflict of interest

The authors declare that the research was conducted in the absence of any commercial or financial relationships that could be construed as a potential conflict of interest.

## Publisher’s note

All claims expressed in this article are solely those of the authors and do not necessarily represent those of their affiliated organizations, or those of the publisher, the editors and the reviewers. Any product that may be evaluated in this article, or claim that may be made by its manufacturer, is not guaranteed or endorsed by the publisher.

## Footnotes

^1^ http://www.genome.jp/kegg/
